# Elevated Pontine and Putamenal GABA Levels in Mild-Moderate Parkinson Disease Detected by 7 Tesla Proton MRS

**DOI:** 10.1371/journal.pone.0030918

**Published:** 2012-01-25

**Authors:** Uzay E. Emir, Paul J. Tuite, Gülin Öz

**Affiliations:** 1 Center for Magnetic Resonance Research, Department of Radiology, University of Minnesota, Minneapolis, Minnesota, United States of America; 2 Department of Neurology, University of Minnesota, Minneapolis, Minnesota, United States of America; University of Medicine and Dentistry of New Jersey, United States of America

## Abstract

**Background:**

Parkinson disease (PD) is characterized by the degeneration of nigrostriatal dopaminergic neurons. However, postmortem evidence indicates that the pathology of lower brainstem regions, such as the pons and medulla, precedes nigral involvement. Consistently, pontomedullary damage was implicated by structural and PET imaging in early PD. Neurochemical correlates of this early pathological involvement in PD are unknown.

**Methodology/Principal Finding:**

To map biochemical alterations in the brains of individuals with mild-moderate PD we quantified neurochemical profiles of the pons, putamen and substantia nigra by 7 tesla (T) proton magnetic resonance spectroscopy. Thirteen individuals with idiopathic PD (Hoehn & Yahr stage 2) and 12 age- and gender-matched healthy volunteers participated in the study. γ-Aminobutyric acid (GABA) concentrations in the pons and putamen were significantly higher in patients (N = 11, off medications) than controls (N = 11, p<0.001 for pons and p<0.05 for putamen). The GABA elevation was more pronounced in the pons (64%) than in the putamen (32%). No other neurochemical differences were observed between patients and controls.

**Conclusion/Significance:**

The GABA elevation in the putamen is consistent with prior postmortem findings in patients with PD, as well as with in vivo observations in a rodent model of PD, while the GABA finding in the pons is novel. The more significant GABA elevation in the pons relative to the putamen is consistent with earlier pathological involvement of the lower brainstem. This study provides in vivo evidence for an alteration in the GABAergic tone in the lower brainstem and striatum in early-moderate PD, which may underlie disease pathogenesis and may provide a biomarker for disease staging.

## Introduction

Parkinson disease (PD) is a progressive neurodegenerative disorder characterized by varying combinations of rest tremor, rigidity, bradykinesia, and postural changes. The major pathologic marker of PD is the degeneration of nigrostriatal dopaminergic neurons which leads to a reduction in dopamine (DA) content within the striatum [1]. While loss of the nigrostriatal dopaminergic neurons represents a hallmark of PD, the pathological involvement in PD is not restricted to these neurons. Thus, recent evidence indicates that caudal brainstem structures are involved in PD pathology even before the nigrostriatal pathology [2]. Several neuropathological studies have reported the degeneration in non-dopaminergic pathways, including serotonin [3] and noradrenaline neurons of the pons [4]. In line with these postmortem findings, the locus coeruleus (LC) of the pons was reported as the most affected extrastriatal area in PD with reduction of ^18^F-dopa uptake, which is thought to reflect the degeneration of neurons in this structure [5], [6]. In addition, recent studies of patients with early PD revealed pontomedullary atrophy by voxel based morphometry [7] and decreased T_1_ in the pontomesencephalic junction, indicating neuronal loss in this area [8].

Motivated by these histological, positron emission tomography (PET) and MRI findings indicative of early functional and structural changes in PD, we sought to gain further insights into biochemical alterations in this disease that are thought to precede structural changes. We utilized in vivo proton magnetic resonance spectroscopy (^1^H MRS) that can non-invasively monitor alterations in neurochemical levels and has been used to study neurochemical alterations in PD with some success [9], [10], [11]. Most prior MRS investigations in PD reported few neurochemicals or their ratios, such as the N-acetylaspartate-to-creatine (NAA/Cr) ratio, measured at 1.5 tesla (T) or 3T. For example, only one prior study investigated pontine neurochemistry in PD during life and reported no difference in the NAA/Cr ratio at 3T between subjects with PD and controls [12]. MRS findings in nigrostriatal structures were variable, most of them reporting no differences in PD vs. controls [9], [10], [13], [14], [15]. On the other hand, MRS studies of animal models of PD at higher magnetic fields found alterations in several metabolites such as the neurotransmitters glutamate (Glu) and γ-aminobutyric acid (GABA) [16], [17]. If similar alterations in neurotransmitter systems are detectable in patients, they could lead to increased understanding of PD pathogenesis and serve as markers of progressive neuronal dysfunction.

Challenges associated with MRS in deep brain nuclei in humans include their location, small size and high iron content [10]. High- and ultra-high field MRS in humans presents additional challenges including short T_2_ values and limitations on the maximum achievable transmit power. Therefore, while the feasibility of quantifying “neurochemical profiles” in the human brain at the ultra-high field of 7T was demonstrated previously [18], [19], such studies have primarily been restricted to superficial volumes-of-interest (VOIs) in the occipital lobe. A recent study demonstrated the feasibility of quantifying six metabolites by 7T MRS in deep brain regions from clinical populations [20]. Subsequently, we overcame challenges associated with quantifying neurochemical profiles of up to 15 metabolites from deep brain regions at 7T, including those that are of interest for PD pathology, such as the pons, the substantia nigra (SN) and the putamen [21]. Therefore, the goal of the current study was to investigate neurochemical alterations in these regions that are thought to be progressively involved in PD pathology with MRS at 7T and specifically to determine if alterations in neurotransmitter levels similar to those observed in animal models of PD would be detectable in patients with mild-moderate PD.

## Methods

### Subjects

Thirteen individuals with mild–moderate PD (6 women and 7 men, 56±10 years old, mean±SD) and 12 age-matched healthy volunteers (7 women and 5 men, 54±8 years old) participated in the study after giving written informed consent using procedures approved by the Institutional Review Board: Human Subjects Committee of the University of Minnesota. Participants were not demented (as assessed by the Mini Mental State Exam and Montreal Cognitive Assessment) and mild-moderate disease severity of patients was established with the Unified Parkinson Disease Rating Scale (UPDRS) and Hoehn & Yahr Staging (H&Y) (Table 1). Patients with PD were off their usual antiparkinsonian medications for 12 hours prior to imaging.

**Table 1:** Demographic and clinical characteristics of patients with PD and control subjects and spectral quality measures.

	Pons		Putamen		Substantia Nigra	
	Control	PD	Control	PD	Control	PD
	(n = 11)	(n = 11)	(n = 11)	(n = 11)	(n = 5)	(n = 5)
Male/Female	5/6	5/6	5/6	6/5	1/4	2/3
Age	52.7±8.7	54.4±9.6	54.0±8.0	55.9±9.8	54.4±11.4	63.0±5.8
Age at PD onset	NA	48.5±9.8	NA	50.3±9.4	NA	58.4±6.2
UPDRS^a^ part III in “off” state	0.1±0.3	30.6±9.2*	0.1±0.3	28.2±8.2*	0.0±0.0	30.8±7.8*
Hoehn & Yahr Stage in “off” state	0.0±0.0	2.0±0.0*	0.0±0.0	2.0±0.0*	0.0±0.0	2.0±0.0*
L-Dopa equivalent dosage (mg/day)	NA	380.4±298.9	NA	342.6±298.2	NA	350.0±395.2
Linewidth (Hz)	12.9±3.0	13.2±2.3	20.5±7.1	19.7±5.9	20.8±4.9	24.6±3.9
SNR^b^	16.2±1.7	15.1±1.9	10.5±2.5	11.0±2.8	7.5±1.2	5.8±1.6

Values given as counts or as mean±SD, as appropriate. NA, not applicable. Statistically significant differences between patient and control groups are marked with.

*p<0.05.

a Unified Parkinson Disease Rating Scale.

b Signal-to-noise ratio of non-weighted spectra calculated by LCModel based on the NAA peak.

### MR Protocol

**Figure 1:**
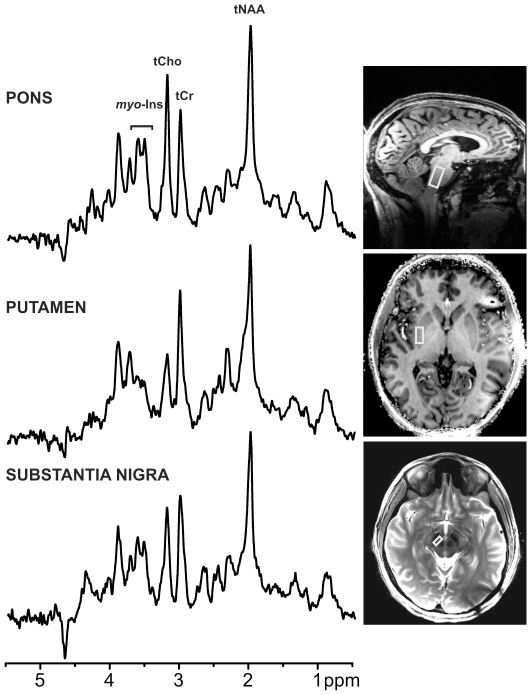
^1^H MR spectra obtained in one patient with PD with STEAM (TR = 5 s, TE = 8 ms) from three VOIs. Processing: Reconstruction of single scan free induction decays (FIDs) from phased array data, frequency and phase correction of FID arrays, FID summation, correction for residual eddy current effects, Gaussian multiplication (σ = 0.1 s), Fourier Transform (FT), zero-order phase correction. Positions of the VOIs are shown on T_1_ – weighted images for pons and putamen and on a T_2_– weighted image for substantia nigra (SN). tNAA, total N-acetylaspartate; tCho, total choline; tCr, total creatine; *myo*-Ins, *myo*-inositol.

MR experiments were performed using a 7T, 90-cm horizontal bore magnet (Magnex) equipped with a Siemens console. A 16-channel transmit/receive transmission line array head coil was used [22]. Images acquired with a 1-mm isotropic resolution MPRAGE sequence (repetition time TR = 3 s, inversion time TI = 1.5 s, echo time TE = 3.67 ms, 192 partition-encode steps, 256 phase-encode steps, 256 data points in the read direction, nominal flip angle = 6°, total acquisition time = 6 min 58 s) were used for the selection of the pons and putamen VOIs. Images acquired with a transverse multislice turbo spin echo sequence (field of view, 180×180 mm^2^; TR = 3 s; TE = 93 ms; flip angle = 150°; slice thickness = 3 mm; 48 slices; one average) were used to select the SN VOI. Destructive B_1_^+^ interferences in the VOI were reduced by localized B_1_^+^ shimming as described before [21]. Spectra were measured with a short-echo stimulated echo acquisition mode (STEAM) sequence (TE = 8 ms, TR = 5 s, mixing time TM = 32 ms) with variable power RF pulses with optimized relaxation delays (VAPOR) water suppression and outer volume saturation [21], [23]. Spectra were acquired from posterior pons (30×10×15 mm^3^, number of transients NT = 128), posterior putamen (12×8×18 mm^3^, NT = 128) and SN (6×13×13 mm^3^, NT = 384) (Fig. 1). Pons and putamen data were acquired in all subjects while SN spectra were acquired from a subset of the volunteers because this acquisition necessitated a second scanning session and only 5 patients and 5 controls were able to participate in a second scan. Pons and putamen data from 11 subjects in each group were included in the final analysis because spectra from two patients and one control volunteer were excluded in each case due to unwanted coherences noted in the spectra. The selection of the pons and putamen VOIs was based on MPRAGE images reconstructed in three orthogonal planes. The boundaries of the VOIs were traced in all slices and their orientation adjusted to ensure that only pontine or putamenal tissue was included in the VOI with minimal partial volume effects. The same procedure was followed with turbo spin echo sequence images to select the SN VOI although partial volume effects could not be fully eliminated even with a ∼1 mL VOI. The putamen and SN VOIs were selected contralaterally to the more severely affected side of patients with PD. The location of the putamenal VOI was chosen based on the known pattern of dopamine depletion in PD. Namely, dopamine depletion starts from the posterior putamen and proceeds gradually to other parts of the striatum based on ^18^F-dopa PET [24]. In addition, postmortem evidence demonstrates more severe dopamine depletion in the posterior than the anterior putamen [25], [26]. The location of the pontine VOI was chosen based on a previous voxel based morphometry study that indicated atrophy in this region [7]. Localizer images were repeated at the end of the measurement from each VOI to confirm negligible gross motion of the volunteer.

Unsuppressed water spectra acquired from the same VOI were used to remove residual eddy current effects and to reconstruct the phased array spectra [27]. Single shot spectra were first averaged over 16 scans prior to frequency alignment to the NAA methyl signal.

### Determination of anatomical structures encompassed by the VOI

To delineate exactly which nuclei were included in our VOI, the MPRAGE and turbo spin echo images were registered by full affine transformations to the MNI152 space (average T_1_ weighted brain image constructed from 152 normal subjects at the Montreal Neurological Institute, Montreal, QC, Canada) using FSL-FLIRT (Oxford Centre for Functional Magnetic Resonance Imaging of the Brain Software (FSL, www.fmrib.ox.ac.uk/fsl) Linear Image Registration Tool) [28]. The registration matrix was then used to transform the VOI data onto the MNI152 space. Coordinates and brain regions encompassed by the VOIs were identified by using Talairach Daemon Labels of FSL [29]. The identification of nuclei included in the posterior pons and SN VOIs was based on visual comparison with schematic drawings of gross anatomy obtained from human brainstem atlas [30], [31] and a template of LC [32].

### Metabolite quantification

Metabolites were quantified using LCModel [33], [34]. The model spectra of alanine (Ala), aspartate (Asp), ascorbate/vitamin C (Asc), glycerophosphocholine (GPC), phosphocholine (PC), creatine (Cr), phosphocreatine (PCr), GABA, glucose (Glc), glutamine (Gln), Glu, glutathione (GSH), *myo*-inositol (*myo*-Ins), lactate (Lac), NAA, N-acetylaspartylglutamate (NAAG), phosphoethanolamine (PE), *scyllo*-inositol (*scyllo*-Ins) and taurine (Tau) were generated based on previously reported chemical shifts and coupling constants [35], [36]. Macromolecule spectra were acquired from the occipital cortex of 5 volunteers using an inversion recovery sequence (TR = 2 s, TI = 0.680 s) [37]. Metabolite concentrations were obtained relative to an unsuppressed water spectrum acquired from the same VOI assuming a water content of 72% for pons, 78% for putamen and 76% for substantia nigra [38], [39]. Concentrations were not corrected for T_1_ and T_2_ effects because long TR and ultra-short TE values were used. Metabolites quantified with Cramér-Rao lower bounds (CRLB, estimated error of the metabolite quantification) >50% were classified as not detected, as suggested by the LCModel manual [40]. As a secondary filter to select reliable metabolite concentrations, only metabolites quantified with CRLB ≤50% in at least half of the spectra from a brain region were reported. This leads to a selection of neurochemicals with average CRLB ≤∼30% (Fig. S1). If the correlation between two metabolites was consistently high (correlation coefficient <−0.5) in a given region, their sum was reported, such as Glc + Tau, NAA + NAAG (tNAA, total NAA), Cr + PCr (tCr, total creatine), GPC + PC (tCho, total choline).

### Statistical Analysis

Statistical analyses were conducted using SPSS (SPSS, Chicago, IL). MRS data from the two groups were compared using a one-way analysis of variance for each metabolite concentration and CRLB in each brain region. Due to the pilot nature of the study p-values shown have not been adjusted for multiple testing. Relationships between clinical scores and metabolite concentrations were evaluated using Pearson correlation coefficients. Measures of spectral quality (signal-to-noise ratio, SNR, and linewidth) and demographic and clinical characteristics (age, UPDRS and H&Y) of the two groups were compared using the two-tailed, unpaired student's t-test.

## Results

Patient and control groups were not different with regards to age and gender (Table 1). All patients had late onset, idiopathic PD and were at H&Y stage 2, considered to be mild-to-moderate disease severity. All but one of the patients were taking L-Dopa or other antiparkinsonian medications that were held for 12 hours prior to the MR scan.

Figure 1 shows representative spectra obtained from the 3 brain regions from a patient with PD. A VOI analysis was performed by transforming each subject's anatomical images to MNI152 space. This approach normalizes brain position and shape and limits variability of regions due to head size and position. Then, the boundaries of VOIs were traced in the MNI space and the brain regions encompassed by the VOIs were identified (Table 2). This analysis showed that multiple noradrenergic and serotonergic nuclei were included in the pons VOI and both pars compacta and pars reticulata were included in the SN VOI.

**Table 2:** The anatomical structures and nuclei encompassed by the three VOIs identified using Talairach Daemon Labels of FSL.

VOI	Brain Regions
Pons	Locus Coeruleus (Noradrenergic center)
	Raphe Nuclei (Serotonergic center)
	Medial Parabrachial Nucleus (Noradrenergic center)
	Pontine Reticular formation
	Abducens Nucleus
	Nucleus Reticularis Centralis
	Facial Nucleus
Putamen	Posterior Putamen
Substantia Nigra	Pars Compacta
	Pars Reticulata
	Ventral Tegmental Area

Artifact free spectra with good SNR and spectral resolution and excellent water suppression were obtained in all brain regions for both patients and controls. The full width at half maximum and SNR values determined by LCModel were not different between patients and controls for any VOI (p>0.05, Table 1), therefore spectral quality in patients with PD and controls was similar. Spectral linewidths were broader in the putamen and SN than those in the pons, indicative of the iron content in these regions (Table 1). The spectral patterns were characteristic of each of the brain regions; note for example the ratio of the creatine and choline peaks in the pons vs. the putamen and SN spectra (Fig. 1).

**Figure 2:**
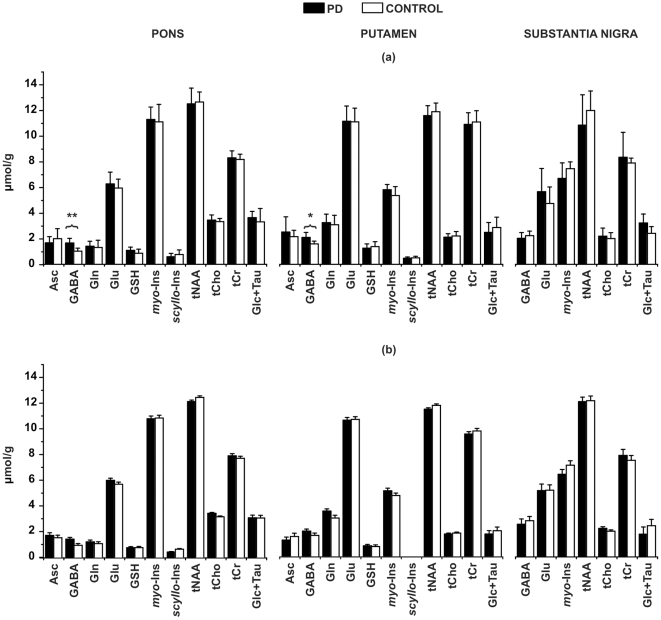
Neurochemical profiles determined by LCModel fitting of (a) individual and (b) averaged spectra from the 3 regions-of-interest in patients with PD and healthy controls. Only metabolites quantified with Cramér-Rao lower bounds (CRLB) ≤50% in at least half of the spectra from a brain region were included in the profiles. Metabolites that were significantly different between the two groups are marked with *p<0.05, **p<0.001 in (a). Error-bars: inter-subject SD in (a) and CRLB expressed as µmol/g in (b). Asc, ascorbate; GABA, γ-aminobutyric acid; Gln, glutamine; Glu, glutamate; GSH, glutathione; *myo*-Ins, *myo*-inositol; *scyllo*-Ins, *scyllo*-inositol; tNAA, total N-acetylaspartate; tCho, total choline; tCr, total creatine; Glc, glucose; Tau, taurine.

**Figure 3:**
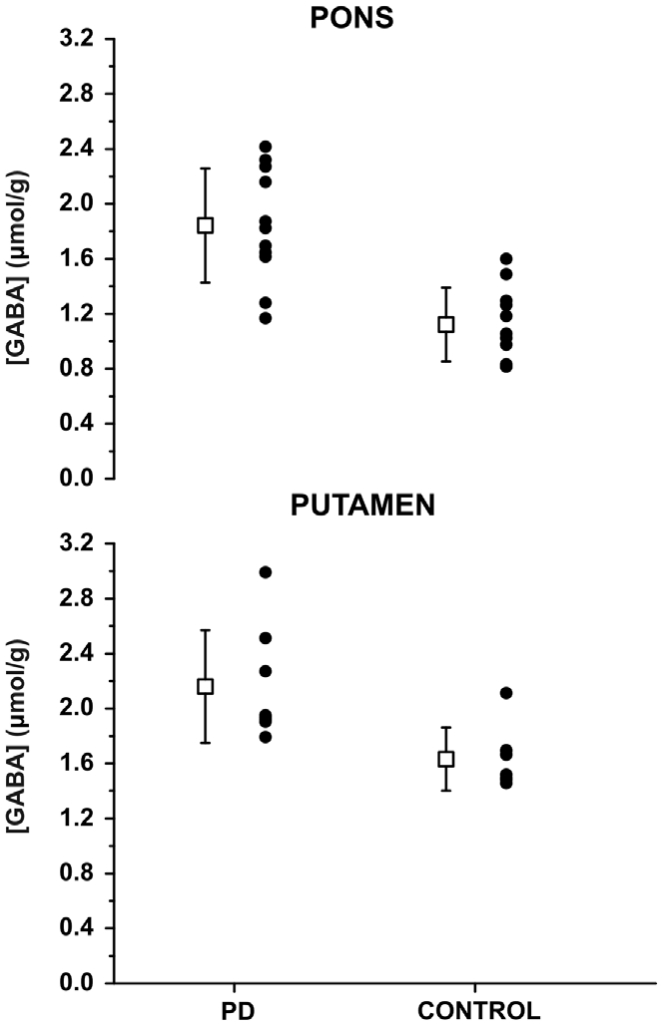
GABA concentrations in pons and putamen by subject groups, together with means (boxes) and standard deviations (error bars).

This spectral quality enabled the quantification of a neurochemical profile consisting of 11 metabolites in the pons and putamen and 7 metabolites in the SN (Fig. 2a). The neurochemical profiles from the SN did not show significant differences between patients and controls. On the other hand, higher GABA concentrations were detected in the pons (p<0.001, 1.6±0.4 µmol/g vs. 1.0±0.2 µmol/g) and putamen (p<0.05, 2.1±0.4 µmol/g vs. 1.6±0.2 µmol/g) in patients with PD relative to controls (Fig. 3). No group differences were observed for the other metabolites (Fig. 2a). To ensure that the quality of individual spectra was sufficient to accurately assess these trends, averaged spectra from the patient and control groups were quantified identically and showed the same trends (Fig. 2b). Consistent with higher GABA concentration in patients, the CRLB of GABA were lower both in the pons (p<0.001, 20.1±5.0 vs. 32.1±6.8%) and putamen (p<0.07, 21.2±5.9 vs. 27.4±6.1%) in patients with PD relative to controls (Fig. S1).

There were no significant relationships between the GABA concentrations and either the UPDRS scores (total and part III) or H&Y scores for any of the VOIs.

## Discussion

Here using ultra-high field MRS we demonstrate elevated GABA levels in the pons and putamen of patients with mild-moderate PD as compared to age- and gender-matched healthy controls. The striatal GABA elevation is consistent with postmortem findings in patients with PD [25] and with in vivo observations in an animal model of PD [16], [17]. The pontine GABA finding is novel. Furthermore, the GABA elevation was greater in the pons (64%) than in the putamen (32%). Data obtained from the SN in a subset of the volunteers revealed no group differences.

**Figure 4:**
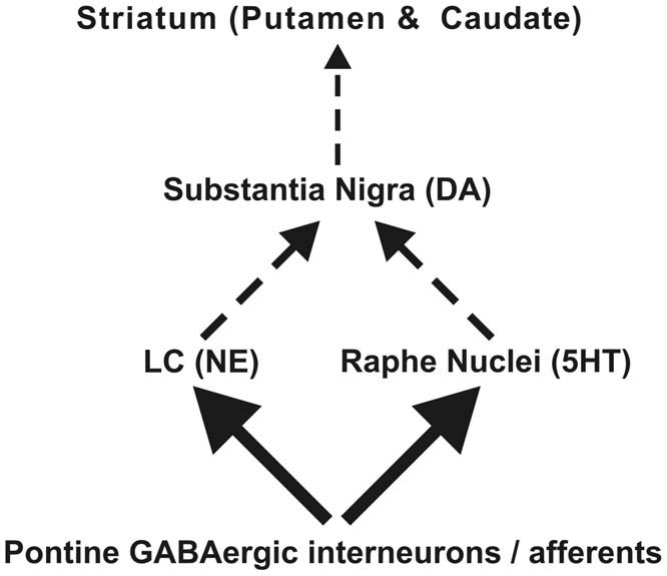
Proposed changes in pontine-nigral-striatal pathways in PD. Enhanced pontine GABAergic activity (wider arrows) onto serotonergic (5HT) locus ceruleus (LC) and/or noradrenergic (NE) raphe neurons could result in a reduction in excitatory outflow (dashed arrows) to substantia nigral neurons. As a result, this may decrease nigral dopaminergic activity to the striatum (indicated by thin dashed arrow).

Since Braak suggested a caudorostral progression of α-synuclein pathology in PD starting in the medulla oblongata [2], [41], only one study has investigated the neurochemistry of the pons in PD with MRS [12]. Ratios of NAA, creatine and choline were investigated in this study, and no differences were seen between patients and controls. Nonetheless, neuronal loss in the pons including the noradrenergic neurons of the LC [4] and the serotonergic neurons of the raphe nucleus [3] is a well documented postmortem finding. Additionally, neuronal loss in the pons has been indicated by several in vivo MRI studies as a reduction in the neuromelanin-related signal localized to the LC [42] and atrophy of the pons/medulla detected by voxel based morphometry [7]. Another recent MRI study reported decreased T_1_ in the pontomesencephalic junction [8] and concluded that gray matter loss was likely the major determinant of this T_1_ decrease. Thus, a neurochemical abnormality was expected in the caudorostral extent of the pons. The most likely neurochemical alteration to indicate neuronal loss would be a lower NAA level [43], which we did not observe, likely due to partial volume effects. On the other hand, we did observe a robust GABA elevation, indicating that this neurochemical difference was more widespread within the pontine VOI. The pontine VOI in this study encompassed some non-dopaminergic brainstem nuclei, such as the right and left LC, the reticular formation and the raphe nuclei (Table 2). It remains to be determined if the GABA abnormality relates to changes in these nuclei. Most likely this change is a result of alterations in several classes of neurons of the brainstem. For instance, others have shown a reduction in ^18^F-dopa uptake in the LC region in patients with more advanced PD (H&Y 2–3) indicating a progressive loss of noradrenergic terminal function [5], [6], [44]. In addition, a reduction in median raphe serotonin 5-hydroxytryptamine receptor 1A binding in PD has been reported [45]. Thus an elevated GABA tone can plausibly contribute to a functional deficit in the LC and raphe nuclei, which are under GABAergic regulation. Therefore, elevated GABA levels in GABAergic interneurons and terminals present in these nuclei [46], [47] may suppress the activity of the noradrenergic and serotonergic neurons that project to the SN. Consequently this would affect the activity of the nigral dopaminergic neurons that project to the striatum (Fig. 4). Taken together, these data suggest that altered GABAergic regulation of non-dopaminergic neurons of the pons may be relevant to PD.

Regarding our putamenal findings, postmortem studies of individuals with advanced disease demonstrated elevated striatal GABA levels, particularly in the putamen [25]. A negative correlation between GABA and DA was observed in the putamen in this postmortem work; suggesting that the striatal GABA elevation was associated with dopaminergic terminal loss [25], [48]. In line with the postmortem findings in patients, a significant increase in GABA concentration was shown in the striatum of the 1-methyl-4-phenyl-1,2,3,6-tetrahydropyridine (MPTP) mouse model of PD [16], [17]. The involvement of striatal GABAergic neurotransmission in PD was further evaluated by quantification of messenger RNA coding for the 67 kDa isoform of glutamic acid decarboxylase (GAD67), one of the two GABA synthesizing enzymes. A significant increase in GAD67 mRNA levels was measured in the putamen of MPTP-treated monkeys and rats relative to controls [49], [50], consistent with elevated GABA concentrations in this region. Interestingly, manganese-exposed workers who are at high risk for PD also show significantly higher GABA levels than controls in a brain region containing the thalamus, putamen and globus pallidus [51]. In the current study we showed that elevated striatal GABA is present *in vivo* in patients with mild-moderate PD. This GABA elevation in the putamen likely stems from striatal GABAergic neurons, rather than GABAergic afferents, because the primary afferents to the striatum are glutamatergic and dopaminergic, while the predominant cell type in the striatum is the medium sized GABAergic spiny neurons. Animal models of PD have shown that experimental lesions of the dopaminergic nigrostriatal pathway result in elevated striatal GABA content [52], which may explain the GABA elevation observed in the putamen in PD. We did not observe a relationship between GABA levels and disease severity based on clinical measures. The lack of a correlation between GABA levels and clinical scores is likely due to the similarity of the clinical stage of our patients, i.e. a small dynamic range (all were at H&Y stage 2), and awaits further investigations with larger groups of patients in a wider range of disease stages. It remains possible that the elevated GABA concentration in the posterior putamen is secondary to a loss of nigrostriatal DA terminals, and therefore may provide complimentary information to the striatal DA depletion detectable by PET scanning [53]. Future cross-sectional and longitudinal investigations of DA by PET and GABA by MRS in parallel could aid in monitoring disease progression. In addition, evaluation of individuals scanned on and off medication may help clarify if the GABA elevation is secondary to DA loss. While we scanned subjects off medications for 12 hours, intermediate- and long-lasting effects of antiparkinsonian medications on the current findings cannot be excluded. Thus medication effects can be limited by studying drug-naïve patients. Prospective ^1^H MRS studies to investigate the consequences of striatal GABA alterations in other regions could also provide further insight. Specifically, investigations of GABA concentrations in the medial pallidal segment and motor thalamic nuclei would be critical since these regions play an important role regarding the outputs of the basal ganglia to the cerebral cortex.

The main limitation of our study is the small sample size. This may have prevented the detection of neurochemical alterations in the SN. Alternatively, the very small size of the VOI (∼1 mL) necessitated by the anatomy of the SN (resulting in low SNR), and high iron content (causing broader intrinsic linewidths, Fig. 1, Table 1) may have obscured neurochemical differences (note the higher standard deviations in SN vs. putamen and pons in Fig. 2a). Similarly, in a prior study of the SN at 4T, we had detected trends, but no statistically significant neurochemical differences, between 10 patients with mild-moderate PD vs. 11 age- and gender-matched healthy controls [10]. The SN VOI utilized in the current study was ∼half of the size of the VOI used in our prior study [10] and also obliqued to better conform with nigral anatomy.

We reported raw p-values here due to the pilot nature of the study, although note that the GABA difference in the pons remains significant after a strict Bonferroni correction for the multiple metabolites measured (significance at p<0.05/11 = 0.0045) and therefore the finding is robust. The difference in the putamen was less significant, which may reflect the earlier involvement of the pons in PD pathogenesis. Alternatively, the CRLB cut-off (CRLB ≤50%) we used to select reliable concentrations may have biased the GABA concentration estimates of controls in the putamen. In the putamen, seven of eleven control subjects' GABA concentrations met our reliable quantification criterion and four subjects' GABA concentrations had CLRB in the range of 50–55% and hence were excluded from the analyses. These excluded GABA concentrations were around ∼1 µmol/g and their inclusion would lower the average GABA concentration in the control group and increase the significance level of the GABA difference in putamen (p<0.001). On the other hand, a larger GABA difference in the pons (52%) vs. the putamen (20%) was supported by evaluation of averaged spectra (Fig. 2b) that overcomes issues with insufficient SNR in spectra from individuals.

Recent developments involving editing techniques have allowed reliable in vivo measurements of GABA without other overlapping resonances in the human brain [54], [55], [56]. In this study, we chose to utilize unedited spectroscopy to quantify a neurochemical profile and to simultaneously assess potential differences in both neuronal and glial markers, such as NAA and *myo*-inositol, respectively, and in neurotransmitter levels, such as glutamate and GABA. Based on the findings of the current study, future studies can utilize edited spectroscopy to focus on measurements of GABA in PD. However, edited MRS measurements of GABA require larger VOI than those utilized here because of longer echo times and consequently lower SNR. Also, edited GABA measurements need to take potential differences in T_2_ between patients and controls into account when interpreting concentration differences. Such potential T_2_ differences are negligible at the ultra-short echo times used in the current study. While T_2_ differences between patients with PD and controls are unlikely for the pons and putamen based on similar linewidths we observed (Table 1), they could potentially confound the quantification of GABA concentration using edited spectroscopy in the SN based on the linewidths we observed in this VOI. Consistently, PD-dependent iron deposition in SN has shown a strong correlation with T_2_ shortening in this region [57].

In conclusion, the present study demonstrated an elevation in pontine and putamenal GABA levels in mild-moderate PD that may underlie aspects of disease pathogenesis and pathophysiology. Whether these are primary or secondary alterations and the impact of treatment on them remain to be determined. These novel findings suggest that further studies with ^1^H MRS may aid in assessing pathogenetic theories of PD and in disease staging together with other noninvasive neuroimaging modalities.

## Supporting Information

Figure S1 Cramér-Rao lower bounds (CRLB) from the 3 regions-of-interest in patients with PD and healthy controls. Only metabolites quantified with CRLB ≤50% in at least half of the spectra from a brain region were included. CRLB of metabolites that were significantly different or showed a trend between the two groups are marked with *p<0.07, **p<0.001. Error-bars: inter-subject SD. Asc, ascorbate; GABA, γ-aminobutyric acid; Gln, glutamine; Glu, glutamate; GSH, glutathione; *myo*-Ins, *myo*-inositol; *scyllo*-Ins, *scyllo*-inositol; tNAA, total N-acetylaspartate; tCho, total choline; tCr, total creatine; Glc, glucose; Tau, taurine.
(TIF)
