# Supplementary figures and images for: Elevated Pontine and Putamenal GABA Levels in Mild-Moderate Parkinson Disease Detected by 7 Tesla Proton MRS
Source: PLoS One. 2012 Jan 25;7(1):e30918. doi: 10.1371/journal.pone.0030918 (PMC3266292; doi:10.1371/journal.pone.0030918)

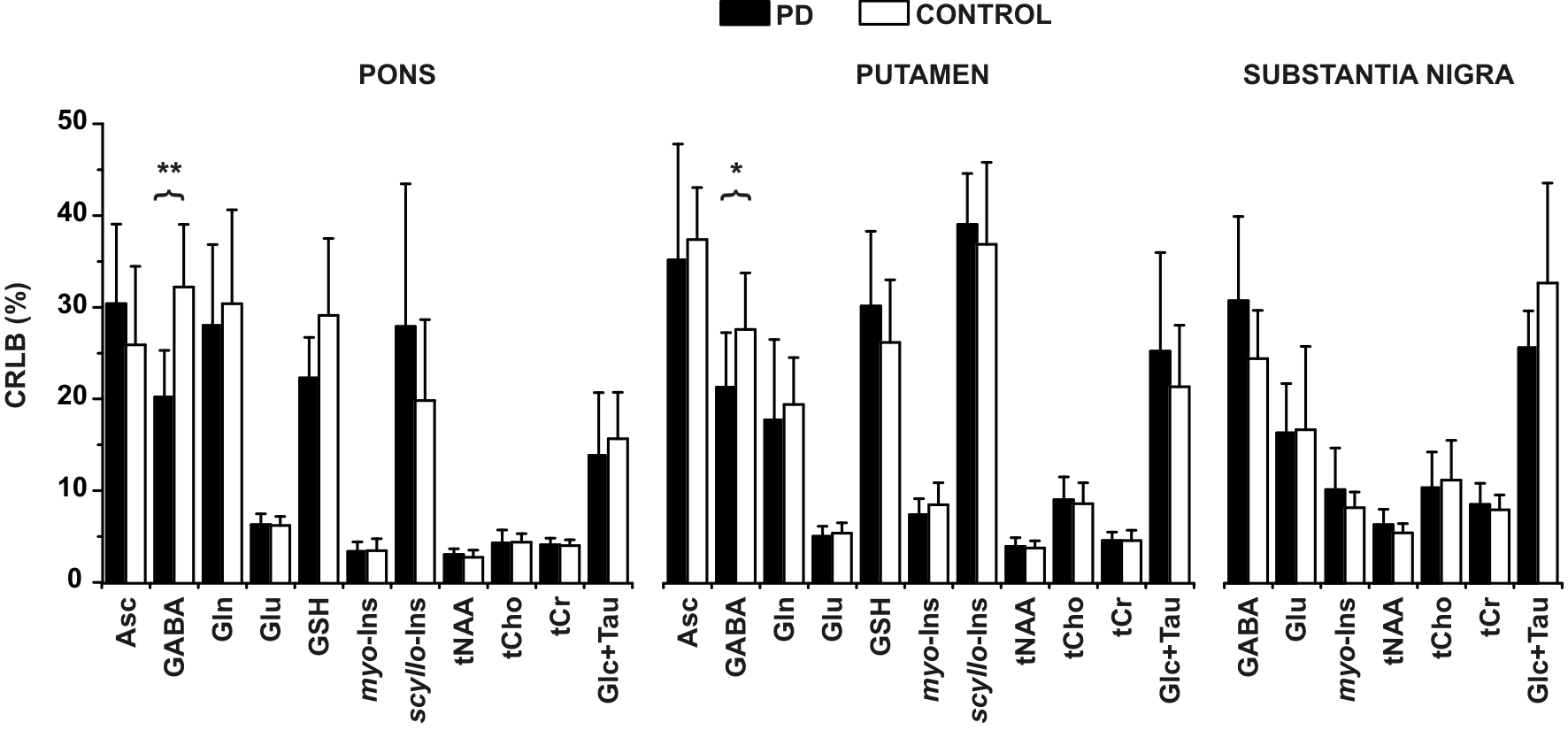

Supplement: Figure S1 — Cramér-Rao lower bounds (CRLB) from the 3 regions-of-interest in patients with PD and healthy controls. Only metabolites quantified with CRLB ≤50% in at least half of the spectra from a brain region were included. CRLB of metabolites that were significantly different or showed a trend between the two groups are marked with *p<0.07, **p<0.001. Error-bars: inter-subject SD. Asc, ascorbate; GABA, γ-aminobutyric acid; Gln, glutamine; Glu, glutamate; GSH, glutathione; myo-Ins, myo-inositol; scyllo-Ins, scyllo-inositol; tNAA, total N-acetylaspartate; tCho, total choline; tCr, total creatine; Glc, glucose; Tau, taurine. (TIF) [file pone.0030918.s001.tif]
